# Allogeneic hematopoietic cell transplantation in an adult patient with Glanzmann thrombasthenia

**DOI:** 10.1002/ccr3.1206

**Published:** 2017-10-05

**Authors:** Ana R. Cid, Pau Montesinos, Isabel Sánchez‐Guiu, Saturnino Haya, Jose I. Lorenzo, Jaime Sanz, Federico Moscardo, Nieves Puig, Dolores Planelles, Santiago Bonanad, Guillermo F. Sanz, Vicente Vicente, Consuelo González‐Manchón, María L. Lozano, José Rivera, Miguel A. Sanz

**Affiliations:** ^1^ Unidad de Hemostasia y Trombosis Servicio de Hematología Hospital Universitario y Politécnico La Fe Valencia Spain; ^2^ Unidad de Trasplante de Células Hematopoyéticas Servicio de Hematología Hospital Universitario y Politécnico La Fe Valencia Spain; ^3^ Servicio de Hematología y Oncología Médica Hospital Universitario Morales Meseguer Centro Regional de Hemodonación Universidad de Murcia, IMIB‐Arrixaca, CIBERER Murcia Spain; ^4^ Centro de Transfusión de la Comunidad Valenciana Valencia Spain; ^5^ Departament Cellular and Molecular Medicine Centro de Investigaciones Biológicas (C.S.I.C.) Madrid Spain

**Keywords:** Adult, antibodies, bleeding, thrombocytopathy, transplantation

## Abstract

Glanzmann thrombasthenia is a rare bleeding disorder that can present life‐threatening bleeding. Our patients develop antiplatelet antibodies that become refractory to any pharmacological treatment. Allogeneic hematopoietic stem‐cell transplantation is the only currently curative procedure, but has major risks mainly in adult; indeed, our patient died.

## Introduction

Glanzmann thrombasthenia (GT) is an autosomal recessive bleeding disorder characterized by either qualitative or quantitative abnormalities of integrin *α*IIb*β*3 as a result of different mutations across genes *ITGA2B* and *ITGB3* 1. Recently, we characterized the largest series of GT in the Iberian Peninsula and identified novel causative mutations 2. Patients often present recurrent and persistent mild mucocutaneous, or even life‐threatening, bleeding, with hypermenorrhea being a major symptom in women. The clinical management of bleeding is generally based on local measures and antifibrinolytic agents for minor hemorrhages, and platelet concentrates (PCs) for major hemorrhages 3. Some GT patients with a severe clinical history who develop antiplatelet antibodies (either anti‐*α*IIb*β*3 or against human leukocyte antigen [HLA]) become refractory to platelet transfusions 4. In some of these patients, administration of recombinant factor VIIa (rFVIIa, NovoSeven; Novo Nordisk A/S, Bagsvaerd, Denmark) could be successful 4, 5. Allogeneic hematopoietic stem‐cell transplantation (HSCT) is considered a curative treatment for this disease, but a balance must be struck between the morbidity–mortality of transplantation and its benefits. To date, several patients with GT have been successfully transplanted 6, 7, 8, 9, 10, 11, 12, 13, 14, 15, 16. Remarkably, all these patients were children, except for one adult patient with GT who underwent HSCT due to a concomitant diagnosis of acute myeloblastic leukemia 11, 17. We present the case of an adult GT female with antiplatelet antibodies and refractoriness to PCs, a poor responder to rFVIIa, who underwent HSCT from her HLA‐identical male sibling. We describe the transplantation features and the long‐term clinical outcome of this patient.

## Case Report

**Figure 1:**
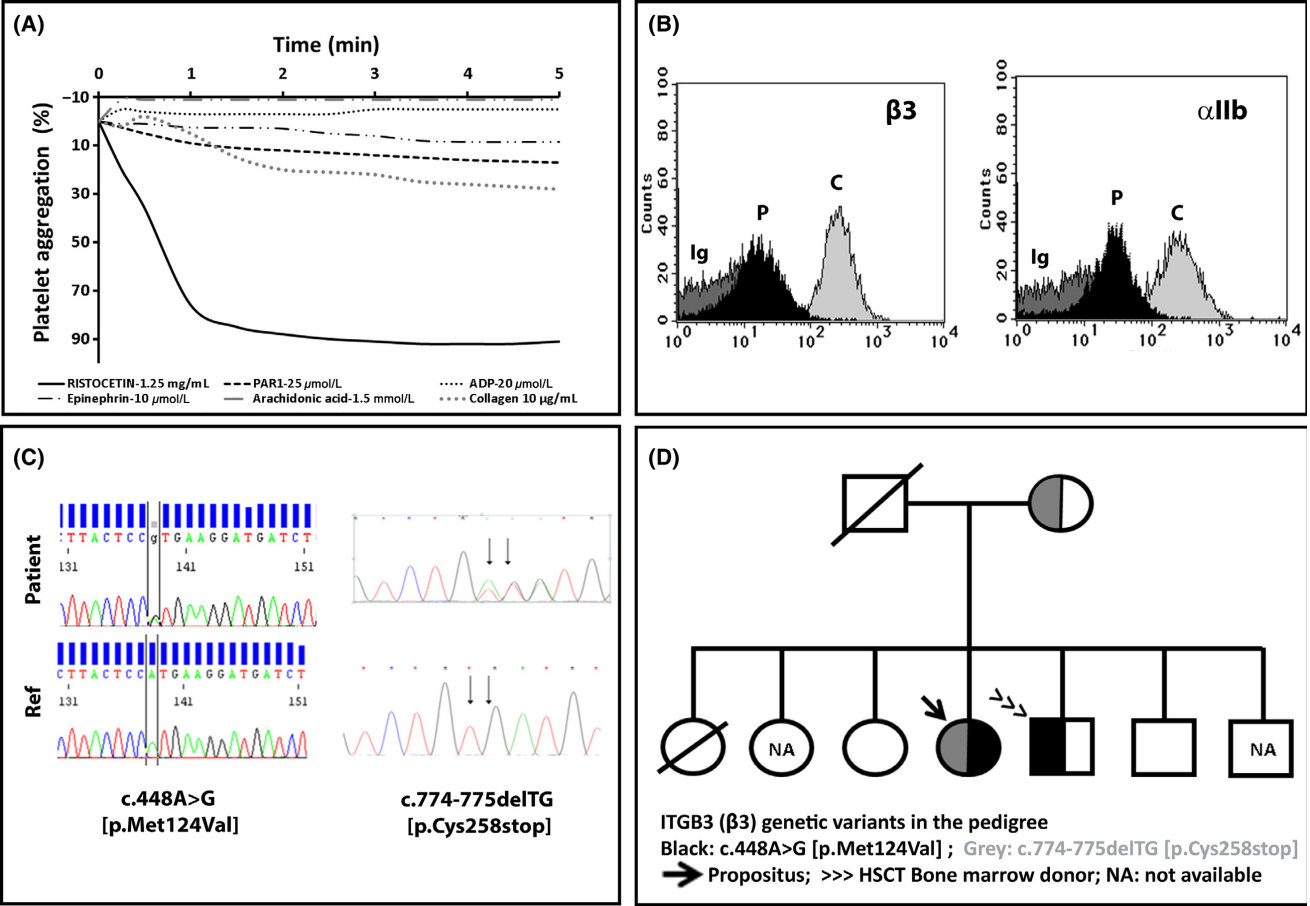
Platelet phenotype and genotype of the index case. The platelet phenotype and genotype of the index case were assessed essentially as described elsewhere 2. (A) Platelet‐rich plasma (PRP) from the index case and a parallel control were prepared from citrated blood, and the platelet aggregation response to different agonists was assessed by standard light transmission aggregometry. The patient displayed abnormal platelet aggregation to all agonists, except ristocetin. A normal response (>90%) to all agonists was found in the control (not shown). (B) The expression of major platelet glycoproteins (GPs) was investigated in PRP from the patient and was controlled by flow cytometry with specific antibodies. Histograms show the severe reduction in *α* IIb*β*3 integrin in the patient (P) compared to the control (C). Ig denotes labeling with an isotype antibody. The expression of other GPs (Ib*α*, IX, and Ia) (not shown) was similar to that of the control. (C) DNA was purified from the patient's blood. All the exon and flanking regions of *ITGB3* and *ITGA2B* were PCR‐amplified with specific oligonucleotides and sequenced by the Sanger method. Mutations c.448A>G and c.774‐775delTG in the *ITGB3* gene were found in the patient. (D) Family pedigree, with identification of carriers of mutations. The patient's father and one sister had died before the study commenced. NA: not available for analysis.

Our female patient was diagnosed with GT at the age of 6. Her family did not present consanguinity, although the death of her sister aged 8 from bleeding after tonsillectomy is noteworthy. Clinically speaking, as an infant she presented unprovoked ecchymosis, epistaxis, gum bleeding, hemorrhages following tooth loss, and intestinal bleeding symptoms which required red blood cells and PCs transfusion. As of menarche, she presented heavy gynecological bleeding that did not respond to therapeutic measures. A complete hysterectomy was performed when she was 25 years old, which was complicated by excess bleeding that required the ligation of hypogastric arteries. Five months later, a bilateral ovarian hematoma required having to remove adnexal remains. Ever since then, her main clinical symptoms have included constant gum bleeding and epistaxis, treated with antifibrinolytics and nasal plugs, and occasionally a PCs transfusion. At the age of 44, excessive bleeding occurred after professional dental cleaning. Development of antiplatelet antibodies was investigated, and presence of anti‐*α*IIb*β*3 and anti‐HLA was confirmed. Since then, her bleeding episodes have been treated with rFVIIa, but her response to this drug was poor. At the age of 47, she was admitted to hospital for persistent hematuria. A cystoscopy was performed, and a double J catheter was placed. However, controlling the hemorrhage proved difficult despite administration of rFVIIa and PCs. Given her poor quality of life with frequent hospitalizations because of bleeding (16 in the 2 years), accompanied by the frequent usage of rFVIIa, she was evaluated for HSCT. Her GT diagnosis was re‐evaluated and confirmed by additional platelet functional studies according to ISTH guidelines 18 (Figure 1). Molecular studies revealed that the patient was a compound heterozygote for two genetic variants in *ITGB3* (c.448A/G [p.Met124Val] and c.774‐775delTG [p.Cys258stop]) 2 (Figure 1). Thus, in February 2012, at the age of 50, a HSCT was performed from her HLA‐identical brother. The donor was a heterozygous carrier of c.448A/G [p.Met124Val] (Figure 1), yet his platelet function analysis displayed no major platelet function abnormalities (not shown). At the baseline, the patient had a HSCT comorbidity index of 2 (depression and anxiety requiring consultation or treatment, and chronic C virus hepatitis with transaminases elevation up to 2 × upper laboratory normal value). The source of hematopoietic progenitors was peripheral blood with partial T‐lymphocyte depletion and positive CD34+ selection to prevent graft‐versus‐host disease (GvHD). Conditioning was performed with a myeloablative BUFLU regime, and GvHD prophylaxis included prednisone and cyclosporine. An immediate post‐transplantation period took place with no relevant incidences, except for a mild urine infection. Platelet (>50 × 10^9^/L) and neutrophil (>0.5 × 10^9^/L) recovery occurred on day +10 and +18, respectively. She had no bleeding problems and platelets showed complete chimerism (97% donor). In spite of partial T‐lymphocyte depletion and CD34+ selection, the patient developed severe chronic (c) GvHD (mild oral and conjunctival, cutaneous grade 2, hepatic 2, and intestinal grade 2), treated with several lines, including the sequential combination of prednisone, oral budesonide, mycophenolate, tacrolimus, thymoglobuline, and extracorporeal photopheresis. As a result, the patient needed up to 22 hospitalizations in 4 years (mainly because of cGVHD and urinary tract infections), and very frequent outpatient visits at the day hospital. Although extensive cGVHD was stable, she was admitted to hospital in July 2016 with pneumonia and sepsis, which resulted in death in July 2016.

## Discussion

The HSCT cases reported to date were carried out in children and young adults with GT and serious bleeding symptoms, both with and without antiplatelet antibodies, using bone marrow, umbilical cord, or peripheral blood stem cells 17. Most of these patients had HLA‐identical relatives, although a few have undergone non‐family‐related donor transplantation 10, 11, 12, 14, 15. Adult patients tend to present higher morbidity and mortality after transplantation than children, including more severe GvHD. In spite of partial T‐cell depletion, the patient developed severe cGvHD accompanied by frequent hospitalizations, use of medical resources, poor quality of life, and death by infectious complications. In view of the patient's outcome, we should highlight that HSCT did improve neither the patient's quality of life nor her life expectancy. The current experience reinforces that in future rare cases of adult patients with GT proposed for HSCT, this indication should be carefully assessed and may only be established when life‐threatening hemorrhages take place. In addition, efficacious strategies to avoid GvHD should be recommended 19.

In summary, while research in the gene therapy area for GT is ongoing 3, HSCT is still the only currently available procedure to cure GT 3, 20. It is indicated in cases with recurrent life‐threatening bleeding complications, particularly if patients are refractory to platelet transfusions. Transplantation should be performed preferably in childhood given the fewer risks of associated complications, mainly GvHD and platelet refractoriness. In adults, HSCT should be assessed on an individual basis and the risk of transplantation complications should be balanced against the risk of bleeding problems of GT and the ability to control bleeding with the available therapy.

## Authorship

ARC and PM: wrote the paper. PM, JIL, JS, FM, and GFS: planned transplantation and performed its monitoring. ISG, JR, MLL, and CGM: performed the platelet functional and molecular studies. All the authors critically reviewed and approved the final version of the paper.

## Conflict of Interest

The authors declare no conflict of interest.
